# Impact of chronic kidney disease stage on morbidity after gastrectomy for gastric cancer

**DOI:** 10.1002/ags3.12441

**Published:** 2021-02-12

**Authors:** Satoshi Suzuki, Shingo Kanaji, Naoki Urakawa, Gosuke Takiguchi, Hiroshi Hasegawa, Kimihiro Yamashita, Takeru Matsuda, Taro Oshikiri, Tetsu Nakamura, Yoshihiro Kakeji

**Affiliations:** ^1^ Division of Gatrointestinal Surgery Department of Surgery Graduate School of medicine Kobe University Kobe Japan

**Keywords:** chronic renal disease, gastrectomy, gastric cancer, glomerular filtration rate

## Abstract

**Aim:**

The outcomes of gastrectomy for gastric cancer in patients at each severity of chronic kidney disease (CKD) remain unknown.

**Methods:**

We retrospectively analyzed the outcomes of 560 patients who underwent distal or total gastrectomy for gastric cancer between 2009 and 2018. We classified the patients into four groups based on estimated glomerular filtration rate: stage 1/2 (normal to mild, n = 375), stage 3a (mild to moderate, n = 122), stage 3b (moderate to severe, n = 43), and stage 4/5 (severe to end‐stage, n = 20) CKD. The relationship between CKD stage and the incidence of postoperative morbidity was analyzed.

**Results:**

CKD was a predictor of overall morbidity independent of age, gender, American Society of Anesthesiologists Performance Status, pulmonary comorbidity, extent of lymphadenectomy, and operation time in a multivariate analysis. The incidences of overall and severe morbidity were significantly increased with CKD progression (both *P < *.001). Compared to stage 1/2 CKD, the odds of overall morbidity were significantly elevated in stage 3a (odds ratio [OR] 1.87*, P = *.007), stage 3b (OR 3.86*, P < *.001), and stage 4/5 (OR 8.60*, P < *.001). The risk of procedure‐related morbidity was strikingly increased in stage 3b (OR 2.93*, P = *.004). The risk of procedure‐unrelated morbidity elevated markedly in stage 3a (OR 2.77*, P = *.001). A significant graded association between CKD progression and overall morbidity was also revealed in elderly patients (*P = *.001).

**Conclusions:**

The severity of CKD predicts the likelihood and type of morbidity after gastrectomy and can guide surgical decision‐making for patients with gastric cancer.

## Graphical abstract

Chronic kidney disease (CKD) progression increases the risk of morbidity following gastrectomy in gastric cancer patients. The risk elevates substantially at moderate CKD with an eGFR ≤ 45 ml/min/1.73 m^2^.

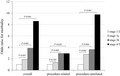

## 1 INTRODUCTION

Gastric cancer is one of the leading causes of cancer deaths throughout the world and continues to increase as populations in developed countries age.^1^ Gastrectomy is the mainstay of treatment for gastric cancer, but its postoperative morbidity impairs the patient's physical condition and often leads to an unfavorable clinical course. Infectious morbidity was also suggested to worsen long‐term outcomes after gastrectomy.^2^, ^3^, ^4^ The identification of a high‐risk candidate who is likely to develop postoperative morbidity is crucial to the planning of surgical treatment for gastric cancer.

Chronic kidney disease (CKD) is increasingly prevalent globally.^5^, ^6^ In Japan, the prevalence rate of end‐stage renal disease is the highest among the world,^7^ and the number of patients with CKD reached 13 million in 2005.^8^ A recent large analysis revealed that the incidence risk of gastric cancer in patients with CKD was significantly higher than in the general population.^9^ Surgery for gastric cancer patients with CKD is expected to increase in the future. Renal dysfunction adversely affects a patient's postoperative course due to impairment of physical function including wound healing, immunity, body fluid regulation, and hemostasis. The presence of end‐stage renal disease has been shown to be accompanied by a higher risk of morbidities after gastroenterological cancer treatment, including gastric cancer treatments.^10^, ^11^, ^12^, ^13^, ^14^, ^15^ However, the impact of mild to moderate CKD on the incidence and type of morbidity after gastrectomy is unclear.^16^

The glomerular filtration rate (GFR) is used in both the definition and the staging of CKD.^17^ The estimated GFR (eGFR) tends to decline with increasing age, and the CKD population based on GFR increases among elderly patients. However, the influence of CKD that is detectable with the use of eGFR in elderly patients undergoing gastrectomy is unknown. We conducted the present study to retrospectively analyze the association between the severity of CKD and the development of morbidity after gastrectomy for gastric cancer, and to elucidate the effect of CKD on surgical outcomes in elderly gastric cancer patients.

## 2 MATERIALS AND METHODS

### 2.1 Patients

From January 2009 to August 2018, a total of 776 patients underwent a gastrectomy for gastric cancer at Kobe University Hospital. Of these, the patients who underwent a proximal gastrectomy, which had less postoperative morbidities in comparison with other type of gastrectomy, and those who received preoperative chemotherapy, which has a potential to increase morbidities, were excluded. We analyzed the cases of the remaining 560 patients who underwent a distal or total gastrectomy with curative intent, using the patients' clinicopathological and treatment data extracted from medical charts. Each patient's preoperative physical status was assessed by age, gender, body mass index (BMI), serum albumin concentration, American Society of Anesthesiologists Performance Status (ASA‐PS), Charlson comorbidity index (CCI), and the presence or absence of diabetes mellitus, pulmonary disease, cardiovascular disease, and a history of anticoagulant agent(s) use. Tumor stage was classified according to the Japanese Classification of Gastric Carcinoma.^18^ Surgical procedures were performed according to the Japanese Gastric Cancer Treatment Guidelines.^19^

Postoperative morbidities diagnosed as grade II or higher on the Clavien‐Dindo classification were defined as procedure‐related or procedure‐unrelated morbidities.^20^ Severe morbidities were defined as morbidities diagnosed as grade III or higher. Procedure‐related morbidities included anastomotic leakage, pancreatic fistula, intra‐abdominal abscess, intra‐abdominal bleeding, anastomotic bleeding, delayed gastric emptying, anastomotic stricture, small bowel obstruction, and wound infection. The procedure‐unrelated morbidities were categorized as follows: cardiovascular morbidity (including arrhythmia, ischemic heart disease, heart failure, and stroke), pulmonary morbidity (including pneumonia, atelectasis, pleural effusion, and edema), other‐site infectious morbidity (including cholecystitis, urinary tract infection, enterocolitis, and catheter‐related infection), and hemostatic morbidity (including venous thromboembolism and intestinal bleeding). All procedures followed were in accordance with the ethical standards of the responsible committee on human experimentation (institutional and national) and with the Declaration of Helsinki.

### 2.2 The classification of CKD severity

Renal function was assessed as the eGFR calculated by the Modification of Diet in Renal Disease Study equation that was modified for a Japanese population as follows: eGFR (ml/min/1.73 m^2^) = 194 × Cr^−1.094^ × age^−0.287^ (×0.739 if female).^21^ CKD was defined as an eGFR < 60 ml/min/1.73 m^2^ for ≥ 3 months according to the 2012 Kidney Disease Improving Global Outcomes (KDIGO) guideline.^17^ Based on the patient's eGFR measured at his or her first visit to our hospital, we classified the patients into the following four groups: stage 1/2 CKD (normal to mild CKD: eGFR ≥ 60 ml/min/1.73 m^2^), stage 3a CKD (mild to moderate CKD: eGFR 45 to < 60 ml/min/1.73 m^2^), stage 3b CKD (moderate to severe CKD: eGFR 30 to < 45 ml/min/1.73 m^2^), and stage 4/5 CKD (severe to end‐stage CKD: eGFR < 30 ml/min/1.73 m^2^).

### 2.3 Statistical analyses

Statistical comparisons among different groups were performed with the Mann‐Whitney *U*‐test and Spearman rank correlation for categorical variables, and with an analysis of variance (ANOVA) or Kruskal‐Wallis test for continuous variables, as appropriate. Potential risk factors for postoperative morbidities were evaluated by a logistic regression analysis. Factors with a probability level ≤ 0.05 were adopted for the subsequent multivariate analysis. Independent risk factors were considered appropriate at a probability level < 0.05. All statistical analyses were performed using JMP statistical software, version 11 (SAS, Cary, NC, USA).

## 3 RESULTS

### 3.1 Clinicopathological characteristics

The study cohort consisted of 415 males (74.1%) and 145 females (25.9%) with the mean age 70.3 ± 10.3 years (range 36‐93 years). The 560 patients were assigned to the stage 1/2 CKD group (n = 375, 67.0%), the stage 3a CKD group (n = 122, 21.8%), the stage 3b CKD group (n = 43, 7.7%), and the stage 4/5 CKD group (n = 20, 3.6%). In the stage 4/5 CKD group, eight patients were on hemodialysis preoperatively. The patient characteristics of the four groups are summarized in Table 1.

**TABLE 1:** Patient characteristics

Variables	Stage ½ (n = 375)	Stage 3a (n = 122)	Stage 3b (n = 43)	Stage 4/5 (n = 20)	*P* value
Age, yrs, mean ± SD	67.5 ± 10.3	74.9 ± 8.4	78.4 ± 8.7	73.8 ± 7.8	<.001
Gender					
Male	267 (71.2)	98 (80.3)	35 (81.4)	15 (75)	.016
Female	108 (28.8)	24 (19.7)	8 (18.6)	5 (25)	
BMI, mean ± SD	22.3 ± 3.2	22.0 ± 3.1	23.0 ± 3.1	23.6 ± 2.3	.089
Albumin, mean ± SD	3.9 ± 0.6	3.8 ± 0.5	3.6 ± 0.7	3.6 ± 0.6	<.001
ASA‐PS					
≤2	346 (92.3)	112 (91.8)	36 (83.7)	6 (30)	<.001
3	29 (7.7)	10 (8.2)	7 (16.3)	14 (70)	
CCI					
≤2	363 (96.8)	118 (96.7)	22 (51.2)	5 (25)	<.001
≥3	12 (3.2)	4 (3.3)	21 (48.8)	15 (75)	
Diabetes mellitus					
Absent	307 (81.9)	93 (76.2)	31 (72.1)	12 (60)	.012
Present	68 (18.1)	29 (23.8)	12 (27.9)	8 (40)	
Pulmonary disease					
Absent	340 (90.7)	109 (89.3)	36 (83.7)	17 (85)	.190
Present	35 (9.3)	13 (10.7)	7 (16.3)	3 (15)	
Cardiovascular disease					
Absent	334 (89.1)	90 (73.8)	31 (72.1)	13 (65)	<.001
Present	41 (10.9)	32 (26.2)	12 (27.9)	7 (35)	
Anticoagulant agent(s)					
Absent	311 (82.9)	87 (71.3)	30 (69.8)	10 (50)	<.001
Present	64 (17.1)	35 (29.8)	13 (30.2)	10 (50)	
Histology					
Differentiated	189 (50.4)	70 (57.4)	27 (62.8)	16 (80)	.008
Undifferentiated	186 (49.6)	52 (42.6)	16 (37.2)	4 (20)	
Tumor invasion					
pT1	219 (58.4)	75 (61.5)	18 (43.9)	11 (55)	.750
pT2	40 (10.7)	9 (7.4)	6 (14.6)	5 (25)	
pT3	56 (14.9)	25 (20.5)	9 (22.0)	2 (10)	
pT4	60 (16)	13 (10.7)	8 (19.5)	2 (10)	
Lymph node metastasis					
pN0	253 (67.5)	84 (68.9)	22 (53.7)	12 (60)	.130
pN1	46 (12.3)	12 (9.8)	6 (14.6)	5 (25)	
pN2	32 (8.5)	16 (13.1)	7 (17.1)	1 (5)	
pN3	44 (11.7)	10 (8.2)	6 (14.6)	2 (10)	
Pathological stage					
I	243 (64.8)	80 (65.6)	20 (48.8)	11 (55)	.340
II	56 (14.9)	18 (14.8)	8 (19.5)	8 (40)	
III	58 (15.5)	22 (18.0)	10 (24.3)	1 (5)	
IV	18 (4.8)	2 (1.6)	3 (7.3)	0 (0)	
Surgical approach					
Open	97 (25.9)	36 (29.5)	20 (46.5)	10 (50)	.004
Laparoscopic	278 (74.1)	86 (70.5)	23 (53.5)	10 (50)	
Type of gastrectomy					
Total	115 (30.7)	36 (29.5)	16 (37.2)	7 (35)	.640
Distal	260 (69.3)	86 (70.5)	27 (62.3)	13 (65)	
Splenectomy	33 (8.8)	6 (4.9)	4 (9.3)	0 (0)	.120
Lymphadenectomy					
≤D1+	220 (58.9)	72 (59.0)	20 (46.5)	8 (40)	.160
≥D2	155 (41.1)	50 (41.0)	23 (53.5)	12 (60)	
Operative time, min					
Median (range)	328 (158‒773)	307.5 (139‒680)	316 (142‒501)	333.5 (167‒702)	.420
Blood loss, mL					
median (range)	60 (5‒4365)	59.5 (5‒1740)	270 (10‒1800)	250 (10‒2250)	.067

Abbreviations: ASA‐PS, American Society of Anesthesiologists Physical Status; BMI, body mass index; CCI, Charlson comorbidity index; SD, Standard deviation.

The stage 1/2 group had a significantly younger mean age and a significantly lower proportion of males compared to the other three groups (*P* < .001 and *P* = .016, respectively). The serum albumin concentrations in the stage 3b and 4/5 groups were significantly lower than those in the stage 1/2 and 3a groups (*P* < .001). Patients with higher ASA‐PS and with higher CCI values were significantly more common in the stage 3b and 4/5 groups than in the stage 1/2 and 3a groups (both *P* < .001). Patients with advanced stage CKD showed significant increases of concomitant diabetes mellitus and cardiovascular disease (*P* = .012 and *P* < .001, respectively), but not pulmonary disease. Patients with stages 1 or 2 were less likely to take anticoagulant agents than those with the other three stages (*P* < .001). The stage 3a, stage 3b, and stage 4/5 groups had a histologically differentiated tumor significantly more frequently than the stage 1/2 group (*P* = .008).

With respect to surgical procedures, laparoscopic‐approach surgery significantly decreased with stage progression (*P* = .004). The extent of gastrectomy or lymph node dissection did not differ among the four groups.

### 3.2 Postoperative morbidity

The patients' CKD stages and postoperative morbidity data are summarized in Table 2. Postoperative morbidity developed in 157 patients (28.0%) after gastrectomy. Among them, 75 patients (13.4%) suffered from severe morbidities. The incidences of overall morbidity and severe morbidity increased significantly in parallel with the CKD stage progression (both *P* < .001). The multivariate analysis revealed that an eGFR < 60 (odds ratio [OR] 1.65, 95% confidence interval [CI] 1. 03‐2.63, *P* = .039), age ≥ 75 years (OR 2.15, 95% CI 1.37‐3.40, *P* < .001), male gender (OR 2.41, 95% CI 1.42‐4.24, *P* = .001) ASA‐PS 3 (OR 3.10, 95% CI 1.60‐5.90, *P* < .001), pulmonary comorbidity (OR 2.13, 95% CI 1.15‐3.91, *P* = .016), D2 lymph node dissection (OR 1.66, 95% CI 1.03‐2.68, *P* = .039), and operation time ≥ 300 min (OR 1.87, 95% CI 1.17‐3.00, *P* = .008) were independently associated with overall morbidity (Table 3).

**TABLE 2:** Postoperative morbidities stratified by CKD stage in whole cohort

Variables	Stage 1/2 (n = 375)	Stage 3a (n = 122)	Stage 3b (n = 43)	Stage 4/5 (n = 20)	*P* value
Overall	80 (21.3)	41 (33.6)	22 (51.2)	14 (70)	<.001
Severe (≥ Grade III)	38 (10.1)	22 (18.0)	9 (20.9)	6 (30)	<.001
Both procedure‐related and ‐unrelated	9 (2.4)	5 (4.1)	2 (4.7)	2 (10)	.085
Procedure‐related					
Overall	59 (15.7)	24 (19.7)	15 (34.9)	7 (35)	.002
Anastomotic leakage	10 (2.7)	6 (4.9)	4 (9.3)	3 (15)	.005
Pancreatic fistula	33 (8.8)	12 (9.8)	9 (20.9)	4 (20)	.035
Intra‐abdominal abscess	3 (0.8)	1 (0.8)	1 (2.3)	0 (0)	.696
Intra‐abdominal bleeding	3 (0.8)	1 (0.8)	0 (0)	1 (5)	.619
Anastomotic bleeding	1 (0.3)	0 (0)	0 (0)	0 (0)	.491
Delayed gastric emptying	4 (1.1)	0 (0)	2 (4.7)	1 (5)	.286
Others	7 (1.9)	7 (5.7)	0 (0)	0 (0)	.370
Procedure‐unrelated					
Overall	28 (7.5)	23 (18.9)	11 (25.6)	9 (45)	<.001
Pulmonary	10 (2.7)	14 (11.5)	6 (14.0)	8 (40)	<.001
Other site infection	11 (2.9)	2 (1.6)	1 (2.3)	2 (10)	.142
Cardiovascular	5 (1.3)	5 (4.1)	2 (4.7)	2 (10)	.007
Venous thromboembolism	3 (0.8)	3 (2.5)	1 (2.3)	0 (0)	.224
Intestinal bleeding	1 (0.3)	1 (0.8)	1 (2.3)	0 (0)	.193
Others	1 (0.3)	4 (3.3)	0 (0)	1 (5)	.014

Note

The data are number (%).

**TABLE 3:** Logistic regression analyses for postoperative morbidity

Variables	Univariate analysis			Multivariate analysis		
	OR	95% CI	*P* value	OR	95% CI	*P* value
Age ≥ 75 years	2.60	1.78‒3.81	<.001	2.15	1.37‒3.40	.001
Male gender	2.53	1.57‒4.22	<.001	2.41	1.42‒4.24	.001
BMI ˂18.5	0.98	0.49‒1.87	.952			
BMI ≥ 25	1.18	0.48‒1.86	.476			
Albumin < 3.5g/dL	2.01	1.30‒3.10	.002	1.19	0.71‒1.97	.511
eGFR < 60 ml/min/1.73m^2^	2.43	1.66‒3.58	<.001	1.65	1.03‒2.63	.039
ASA‐PS 3	3.86	2.23‒6.75	<.001	3.10	1.60‒5.90	<.001
CCI ≥ 3	3.00	1.67‒5.40	<.001	1.01	0.49‒2.12	.972
Diabetes mellitus	1.52	0.98‒2.34	.062			
Pulmonary disease	2.52	1.45‒4.34	.001	2.13	1.15‒3.91	.016
Cardiovascular disease	2.16	1.35‒3.43	.002	1.57	0.81‒3.03	.177
Anticoagulant agents	1.59	1.03‒2.43	.038	1.18	0.65‒2.20	.595
pStage II,III,IV	1.53	1.05‒2.24	.027	1.41	0.91‒2.18	.123
Open approach surgery	2.20	1.49‒3.25	<.001	1.09	0.57‒2.09	.799
Total gastrectomy	1.91	1.29‒2.80	.001	1.43	0.89‒2.28	.135
Splenectomy	1.76	0.91‒3.32	.090			
D2 Lymph node dissection	2.03	1.40‒2.96	<.001	1.66	1.03‒2.68	.039
Operation time ≥ 300 min	1.95	1.32‒2.91	<.001	1.87	1.17‒3.00	.008
Blood loss ≥ 300 ml	2.46	1.65‒3.65	<.001	1.37	0.73‒2.56	.323

Abbreviations: ASA‐PS, American Society of Anesthesiologists Physical Status; BMI, body mass index; CCI, Charlson comorbidity index; CI, confidence interval; eGFR, estimate glomerular filtration rate; OR, odds ratio.

**FIGURE 1:**
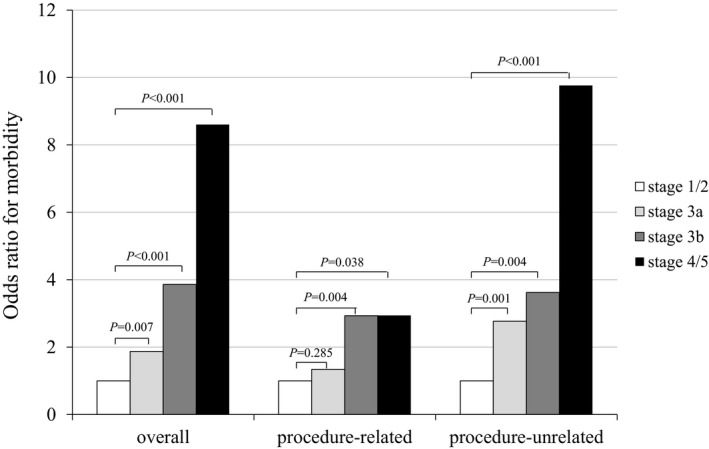
Morbidities at each CKD stage. The odds ratio for procedure‐related, procedure‐unrelated, and overall morbidity at each CKD stage is compared

The odds of overall morbidity were elevated in the stage 3a group (OR 1.87, 95% CI 1.19‐2.92, *P* = .007), stage 3b group (OR 3.86, 95% CI 2.02‐7.42, *P* < .001), and stage 4/5 group (OR 8.60, 95% CI 3.34‐24.95, *P* < .001) compared to the stage 1/2 CKD group (Figure 1). The incidences of overall procedure‐related morbidity, anastomotic leakage, and pancreatic fistula increased with CKD stage progression (*P* = .002, *P* = .005, and *P* = .035, respectively). The odds of procedure‐related morbidity, relative to stage 1/2 CKD, were 1.34 for stage 3a (95% CI 0.78‒2.24, *P* = .285), 2.93 for stage 3b (95% CI 1.44‒5.76, *P* = .004), and 2.94 for stage 4/5 (95% CI 1.07‒7.51, *P* = .038). The odds elevated sharply from stage 3a to stage 3b CKD.

The incidences of overall procedure‐unrelated morbidity, pulmonary events, and cardiovascular events rose with CKD stage progression (*P* < .001, *P* < .001, and *P* = .007, respectively). The odds of procedure‐unrelated morbidity, relative to stage 1/2 CKD, were 2.77 for stage 3a (95% CI 1.52‐5.00, *P* = .001), 3.62 for stage 3b (95% CI 1.56‐7.88, *P* = .004), and 9.76 for stage 4/5 (95% CI 3.67‐25.55, *P* < .001). The odds elevated substantially from stage 1/2 to stage 3a CKD.

### 3.3 Elderly patients

Elderly patients (i.e. those aged ≥ 75 years) accounted for 35% (n = 196) of the total cohort, and 54.6% (n = 107) of the elderly patients had CKD. Overall morbidity significantly increased with CKD stage progression among the elderly patients (*P* = .01, Table 4). Both procedure‐related and procedure‐unrelated morbidities were significantly associated with CKD stage progression in this patient group (*P* = .047 and *P* = .007, respectively). Severe morbidity showed a tendency to increase with CKD stage progression, but the difference did not reach significance.

**TABLE 4:** Postoperative morbidities stratified by CKD stage in the elderly

Variables	Stage 1/2 (n = 89)	Stage 3a (n = 65)	Stage 3b (n = 30)	Stage 4/5 (n = 12)	*P* value
Overall	27 (30.3)	28 (43.1)	16 (53.3)	9 (75)	.001
Severe (≥ Grade III)	11 (12.4)	12 (18.5)	6 (20)	3 (25)	.148
In‐hospital death	2 (2.2)	0 (0)	1 (3.3)	0 (0)	.692
Procedure‐related					
Overall	16 (18.0)	14 (23.1)	8 (26.7)	6 (50)	.047
Anastomotic leakage	3 (3.4)	4 (6.2)	3 (10.3)	2 (16.7)	.062
Pancreatic fistula	6 (6.7)	5 (7.7)	4 (13.3)	4 (33.3)	.040
Procedure‐unrelated					
Overall	13 (14.6)	19 (29.2)	9 (30)	5 (41.7)	.007
Pulmonary	5 (5.6)	11 (16.9)	6 (20)	4 (33.3)	.002
Cardiovascular	1 (1.1)	5 (7.7)	1 (3.3)	1 (6.7)	.121

## 4 DISCUSSION

As most previous articles merely reported that the incidence of morbidity after gastrectomy increased in gastric cancer patients with CKD, the effects of the severity of CKD on what type of morbidity remain unclear. To our knowledge, the present study is the first report that demonstrates a graded association between distinct CKD stages and the incidence and type of morbidity after gastrectomy. A possible explanation for the strong positive correlation between CKD progression and adverse outcomes is that the severity of CKD may reflect a patient's health impairment as well as his or her renal dysfunction. We observed that CKD stage progression was associated with higher ASA‐PS, higher CCI values, and the presence of comorbidities. This finding is in accord with a report demonstrating that frailty was strongly associated with all stages of CKD.^22^ Moreover, CKD is considered to be a cause of sarcopenia, and malnutrition is often concurrent.^23^, ^24^, ^25^, ^26^ Considering these findings, the progression of renal dysfunction in concert with health impairment may contribute to increased postoperative morbidity. ASA‐PS, which reflects a patient's health condition, has been well established as a reliable surrogate for preoperative risk.^27^, ^28^, ^29^ ASA‐PS is assessed subjectively, and only 16.3% of patients were classified with ASA‐PS of 3 or higher in our stage 3b CKD group. As renal function declines, accumulated uremic toxins are thought to lead to inflammation, immune dysfunction, bacterial translocation, atherosclerosis, platelet dysfunction, and altered drug metabolism.^30^, ^31^The severity of a patient's CKD may reflect the patient's vulnerability objectively and may have the potential to predict postoperative morbidity sensitively in comparison with ASA‐PS.

Our present results confirm and extend the findings of a previous investigation in which anastomotic leakage, pulmonary morbidities, and cardiovascular morbidities were frequently observed in patients with stage 3 or severe CKD.^11^ Our findings about graded association further demonstrated that the increase in the risk of postoperative morbidity with CKD progression is not linear, and varies with the type of morbidities. Stage 3 CKD (mild to severe renal dysfunction) was subdivided in the updated guidelines into stage 3a (mild to moderate dysfunction) and stage 3b (moderate to severe dysfunction), based on the risks of mortality and other outcomes.^17^ A significant risk of procedure‐related morbidity was not confirmed in stage 3a CKD by our analyses, but a risk of procedure‐unrelated morbidity was detected. These findings cannot be fully explained. The prevalence rates of comorbidities such as diabetes mellitus, cardiovascular disease, and the use of anticoagulant agents (all of which are likely to strongly affect developing pulmonary and cardiovascular morbidities) were more frequent in our stage 3a and severe CKD groups compared to those in the stage 1/2 CKD group. The development of systemic morbidity may be highly susceptible to impairments of physical status and may be affected by earlier phases of renal dysfunction. On the other hand, procedure‐related morbidities, such as anastomotic leakage and pancreatic fistula, may be directly affected by local conditions, such as impaired wound healing due to decreased blood flow and tissue edema due to atherosclerosis and hypoalbuminemia, with advanced CKD. These findings may provide helpful information to surgeons regarding the indications for modified surgery, such as limited lymphadenectomy, or for the shortening of operation time, to prevent postoperative morbidity, especially for patients with more than moderate CKD. Several studies reported the efficacy of preoperative nutritional support for the patients who underwent gastrectomy in clinical trial. On the other hand, the restriction of protein intake is recommended for control CKD progression. Excessive ingestion of protein has a possibility for decline in eGFR and the progression of CKD stage shows an increased risk of death. Therefore, nutritional intervention for CKD patients has a dilemma for prevention of morbidity and CKD progression. The efficacy of nutritional therapy with protein restriction for CKD patients who undergoing gastrectomy is uncertain.

The severity of CKD is defined by the GFR value. The GFR tends to decline with aging. In the present patient cohort, the proportion of CKD patients among elderly patients was much higher than that in non‐elderly patients (54.6% vs 21.4%). It is doubtful whether a decline in the GFR represents CKD in the elderly. CKD is known to increase the all‐death rate and the rate of death due to cardiovascular causes in the elderly as well as the non‐elderly, and a decline in the GFR is an independent risk factor for cardiovascular death separate from age.^32^ Similarly, CKD was identified as a risk factor for developing postoperative morbidity independently of age in our multivariate analysis. We also observed a graded association between CKD stage progression and postoperative morbidity in elderly patients. GFR‐based CKD can be a graded risk factor for adverse outcomes after gastrectomy across age groups.

This retrospective study at a single institution had several limitations. First, the number of patients was relatively small, and there were a small number of stage 4/5 CKD patients, resulting in limited statistical power. Secondly, our cohort included diverse patients with various physical conditions and disease statuses other than CKD. The frailer patients tended to undergo less invasive procedures, which affect the development of postoperative morbidities. The confirmation of these findings requires a larger study, but our results provide a strategic concept for gastric cancer surgery in CKD patients.

In conclusion, the severity of CKD can be used to predict the likelihood and type of morbidity after gastrectomy across age categories. The risk for procedure‐unrelated morbidity was evident even at the mild stage of CKD. The risk for both procedure‐related and ‐unrelated morbidities increased notably at the moderate stage of CKD with an eGFR < 45 ml/min/1.73 m^2^. CKD progression for the risk stratification of patients undergoing gastric cancer surgery can guide the decision of surgical procedures.

## CONFLICT OF INTEREST

Authors declare no conflict of interests for this article.

## ETHICAL APPROVAL

The protocol for this research project has been approved by a suitably constituted Ethics Committee of the institution and it conforms to the provisions of the Declaration of Helsinki. Institutional Review Board of the Graduate School of Medicine, Kobe University. Approval No. B200084.
